# Supplementary material for: The Small RNA Universe of Capitella teleta
Source: Front Mol Biosci. 2022 Feb 25;9:802814. doi: 10.3389/fmolb.2022.802814 (PMC8915122; doi:10.3389/fmolb.2022.802814)
Supplement: Supplementary file 1 [file DataSheet1.ZIP › Supplement/known/CAPTEscaffold_140_8148.pdf]

Provisional ID	:	CAPTEscaffold_140_8148
Score total	:	16747.3
Score for star read(s)	:	3.9
Score for read counts	:	16740.7
Score for mfe	:	1.7
Score for randfold	:	1.6
Score for cons. seed	:	-0.6
Total read count	:	32848
Mature read count	:	29867
Loop read count	:	0
Star read count	:	2981

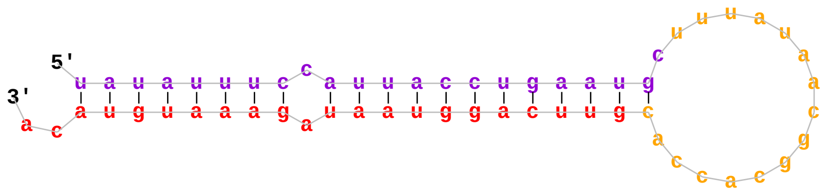

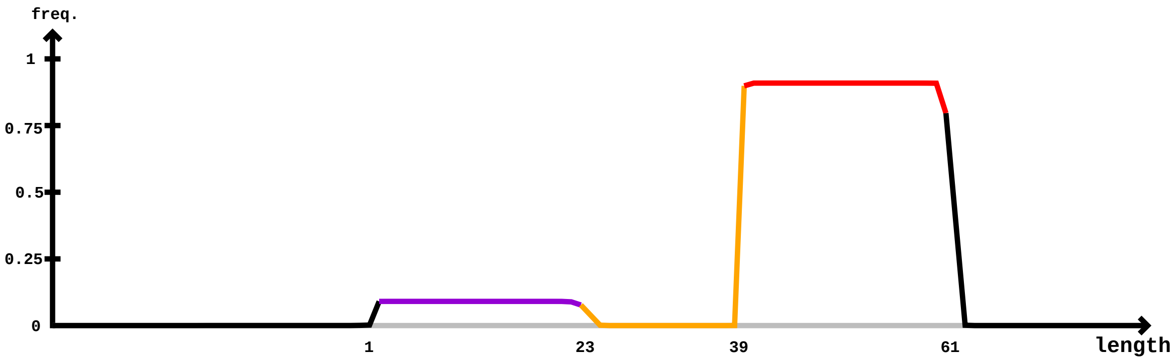

Star Mature

```
5'- aggaagagaagacaauuuguguuuaagcgcucuauauuuccauuaccugaaugcuuuauaacggcaccacguucagguaauagaaauguacagugcuuguacaaaucucgac   -3'   obs
    aggaagagaagacaauuuguguuuaagcgcucuauauuuccauuaccugaaugcuuuauaacggcaccacguucagguaauagaaauguacagugcuuguacaaaucucgac         exp
    aggaagagaagacaauuuguguuuaagcgcucuauauuuccauuaccugaaugcuuuauaacggcaccacguucagguaauagaaauguacagugcuuguacaaaucucgac         known
    .....((((......((((((..((((((((.((((((((.(((((((((((...............))))))))))).)))))))).))))))))))))))))))......     reads     mm     sample
    ..............auuuguguuuaagcgcuc................................................................................     3         0      seq
    ..............................ucuauauuuccauuaccuga..............................................................     2         0      seq
    ..............................ucuauauuuccauuaccugaa.............................................................     2         0      seq
    ..............................ucuauauuuccauuaccugaau............................................................     17        0      seq
    ...............................cuauauuuccauuaccugaau............................................................     7         0      seq
    ...............................cuauauuuccauuaccugaaug...........................................................     26        0      seq
    ...............................cuauauuuccauuaccugaaugA..........................................................     5         1      seq
    ................................uauauuuccauuaccugaa.............................................................     2         0      seq
    ................................uauauuuccauuaccugaau............................................................     31        0      seq
    ................................Aauauuuccauuaccugaau............................................................     1         1      seq
    ................................uauauuuccauAaccugaaug...........................................................     1         1      seq
    ................................Aauauuuccauuaccugaaug...........................................................     1         1      seq
    ................................uauauuucAauuaccugaaug...........................................................     1         1      seq
    ................................uauauuuccauuGccugaaug...........................................................     1         1      seq
    ................................uauauuuccGuuaccugaaug...........................................................     1         1      seq
    ................................Nauauuuccauuaccugaaug...........................................................     1         1      seq
    ................................uauauuuccauuaccugaaug...........................................................     354       0      seq
    ................................uauauuuccauuaccAgaaug...........................................................     1         1      seq
    ................................uauauuuccaGuaccugaaugc..........................................................     1         1      seq
    ................................uauauuuccauuaccugGaugc..........................................................     1         1      seq
    ................................uauauuAccauuaccugaaugc..........................................................     2         1      seq
    ................................Aauauuuccauuaccugaaugc..........................................................     5         1      seq
    ................................uauauuuccauuaccugaaCgc..........................................................     1         1      seq
    ................................uauauuuccauuaccugaauUc..........................................................     1         1      seq
    ................................uauauuuccauuGccugaaugc..........................................................     11        1      seq
    ................................Nauauuuccauuaccugaaugc..........................................................     1         1      seq
    ................................uauauuuccauuaccugaaugc..........................................................     2080      0      seq
    ................................uauaAuuccauuaccugaaugc..........................................................     1         1      seq
    ................................uauauuuUcauuaccugaaugc..........................................................     1         1      seq
    ................................uauauuucUauuaccugaaugc..........................................................     2         1      seq
    ................................uauauuuccauuUccugaaugc..........................................................     1         1      seq
    ................................uauauuucAauuaccugaaugc..........................................................     1         1      seq
```

Star Mature

```
aggaagagaagacaauuuguguuuaagcgcucuauauuuccauuaccugaaugcuuuauaacggcaccacguucagguaauagaaauguacagugcuuguacaaaucucgac
................................uauauuuccauuaccuAaaugc..........................................................  1      1   seq
................................uauauuuccauuaccAgaaugc..........................................................  2      1   seq
................................Gauauuuccauuaccugaaugc..........................................................  2      1   seq
................................uauauuuccauuaccugaaAgc..........................................................  2      1   seq
................................uauauuuccaAuaccugaaugc..........................................................  2      1   seq
................................uaAauuuccauuaccugaaugc..........................................................  2      1   seq
................................uauauuuccauuaccugaaugA..........................................................  14     1   seq
................................uauauAuccauuaccugaaugc..........................................................  1      1   seq
................................uauauuuccauuaccugaauCcu.........................................................  1      1   seq
................................Aauauuuccauuaccugaaugcu.........................................................  2      1   seq
................................uauauuuccaAuaccugaaugcu.........................................................  1      1   seq
................................uauauuuccauuaccugaaugcu.........................................................  265    0   seq
................................uauauuuccauuaccugaaugcC.........................................................  39     1   seq
................................uauauuuccauuaccugaaugcG.........................................................  1      1   seq
................................uauauuuccauuaccugaaugcA.........................................................  36     1   seq
................................uauauuucUauuaccugaaugcuu........................................................  1      1   seq
................................uauauuuccauuaccugaaugcuu........................................................  36     0   seq
................................uauauuuccauuaccugaaugcCu........................................................  3      1   seq
................................uauauuuccauuaccugaaugcuuu.......................................................  1      0   seq
.................................auauuuccauuaccugaaugcu.........................................................  3      0   seq
..................................uauuuccauuaccugaaugcuA........................................................  3      1   seq
.....................................................................cguucagguaauagaaauguac.....................  3      0   seq
.....................................................................cguucagguaauagaaauguaca....................  2      0   seq
......................................................................guucagguaauagaaaugu.......................  3      0   seq
......................................................................guucagguaauagaaaugua......................  12     0   seq
......................................................................guuUagguaauagaaauguac.....................  1      1   seq
......................................................................guucGgguaauagaaauguac.....................  1      1   seq
......................................................................Auucagguaauagaaauguac.....................  2      1   seq
......................................................................gNucagguaauagaaauguac.....................  1      1   seq
......................................................................guuAagguaauagaaauguac.....................  1      1   seq
......................................................................guucaAguaauagaaauguac.....................  1      1   seq
......................................................................guucagguaauagaaauguac.....................  3601   0   seq
......................................................................guucagguGauagaaauguac.....................  3      1   seq
......................................................................guucagguaauagaaaugAac.....................  3      1   seq
......................................................................guucagguaauagaaauguaA.....................  2      1   seq
......................................................................guucagguaauagaaaAguac.....................  1      1   seq
......................................................................gCucagguaauagaaauguac.....................  1      1   seq
......................................................................guucagguaauaAaaauguac.....................  3      1   seq
......................................................................guucagguaauUgaaauguac.....................  1      1   seq
......................................................................gAucagguaauagaaauguac.....................  2      1   seq
......................................................................guucagguaauagUaauguac.....................  2      1   seq
......................................................................guucagguaauagGaauguac.....................  2      1   seq
......................................................................guucagguUauagaaauguac.....................  1      1   seq
......................................................................guAcagguaauagaaauguac.....................  1      1   seq
......................................................................guucaggAaauagaaauguac.....................  1      1   seq
......................................................................Cuucagguaauagaaauguac.....................  1      1   seq
......................................................................guucaggCaauagaaauguac.....................  1      1   seq
......................................................................gGucagguaauagaaauguac.....................  1      1   seq
......................................................................guucagguaauagaaauguUc.....................  1      1   seq
......................................................................Uuucagguaauagaaauguac.....................  6      1   seq
......................................................................guucagguaauagaaaCguaca....................  1      1   seq
......................................................................guucagguaauGgaaauguaca....................  2      1   seq
......................................................................guuUagguaauagaaauguaca....................  27     1   seq
......................................................................guuAagguaauagaaauguaca....................  11     1   seq
......................................................................guucagguGauagaaauguaca....................  10     1   seq
......................................................................guucagguaauagUaauguaca....................  2      1   seq
......................................................................guucagguaauagaaaugAaca....................  4      1   seq
......................................................................gAucagguaauagaaauguaca....................  21     1   seq
......................................................................guucagguaauagaaauUuaca....................  3      1   seq
......................................................................guucGgguaauagaaauguaca....................  6      1   seq
......................................................................guucagguaauagaUauguaca....................  1      1   seq
......................................................................guucagguaauaCaaauguaca....................  2      1   seq
......................................................................guucagguaauagaGauguaca....................  4      1   seq
......................................................................guucagguaauagaaauguaca....................  25093  0   seq
......................................................................guucagguaauagaaaAguaca....................  7      1   seq
......................................................................guucagguaauagaaauguUca....................  6      1   seq
......................................................................guucagguaauagaaauAuaca....................  6      1   seq
......................................................................guucaAguaauagaaauguaca....................  13     1   seq
......................................................................Cuucagguaauagaaauguaca....................  10     1   seq
......................................................................guucaCguaauagaaauguaca....................  3      1   seq
```

Star **Mature**

```
aggaagagaagacaauuuguguuuaagcgcucuauauuuccauuaccugaaugcuuuauaacggcaccacguucagguaauagaaauguacagugcuuguacaaaucucgac
......................................................................guucaUguaauagaaauguaca....................	4	1	seq
......................................................................guucagguaaAagaaauguaca....................	10	1	seq
......................................................................guucagguaauagaaauCuaca....................	1	1	seq
......................................................................guuGagguaauagaaauguaca....................	1	1	seq
......................................................................guucagAuaauagaaauguaca....................	2	1	seq
......................................................................Nuucagguaauagaaauguaca....................	3	1	seq
......................................................................gCucagguaauagaaauguaca....................	4	1	seq
......................................................................guucagUuaauagaaauguaca....................	2	1	seq
......................................................................guucagguaUuagaaauguaca....................	2	1	seq
......................................................................guucagguaauagaaauguaUa....................	5	1	seq
......................................................................guucagguaauUgaaauguaca....................	3	1	seq
......................................................................guucUgguaauagaaauguaca....................	1	1	seq
......................................................................Auucagguaauagaaauguaca....................	4	1	seq
......................................................................guucaggCaauagaaauguaca....................	2	1	seq
......................................................................guucagguaauaAaaauguaca....................	15	1	seq
......................................................................guucagguaauagaaauguacG....................	4	1	seq
......................................................................gGucagguaauagaaauguaca....................	5	1	seq
......................................................................guucagguaGuagaaauguaca....................	2	1	seq
......................................................................guucagguaauagaaUuguaca....................	4	1	seq
......................................................................guucagguaauagaaGuguaca....................	1	1	seq
......................................................................guucagguaauagaaaugCaca....................	4	1	seq
......................................................................guucagguaauaUaaauguaca....................	2	1	seq
......................................................................guucagguaauagaaauguaGa....................	4	1	seq
......................................................................guAcagguaauagaaauguaca....................	26	1	seq
......................................................................Uuucagguaauagaaauguaca....................	49	1	seq
......................................................................guucagguaauagaaauguGca....................	4	1	seq
......................................................................guucagguaauagaaauguaAa....................	5	1	seq
......................................................................guucagguaauagGaauguaca....................	3	1	seq
......................................................................guCcagguaauagaaauguaca....................	2	1	seq
......................................................................guucagguaauagaaauguacU....................	430	1	seq
......................................................................guucagCuaauagaaauguaca....................	1	1	seq
......................................................................guucaggAaauagaaauguaca....................	19	1	seq
......................................................................guucagguaauagaaauguacaU...................	2	1	seq
......................................................................guucagguaauagaaauguacaA...................	7	1	seq
.......................................................................uucagguaauagaaauguac.....................	36	0	seq
.......................................................................uucagguaauagaaUuguac.....................	1	1	seq
.......................................................................uuAagguaauagaaauguac.....................	1	1	seq
.......................................................................uAcagguaauagaaauguac.....................	2	1	seq
.......................................................................Gucagguaauagaaauguac.....................	1	1	seq
.......................................................................uucagguaauagaaauguaca....................	220	0	seq
.......................................................................uucagguaaAagaaauguaca....................	1	1	seq
.......................................................................uucagguaauagaaauguacag...................	38	0	seq
.......................................................................uucagguaauagaaauguacaA...................	3	1	seq
.......................................................................uucagguaauagaaauguacagu..................	7	0	seq
.......................................................................uucagguaauagaaauguacagA..................	28	1	seq
........................................................................ucagguaauagaaauguacagu..................	3	0	seq
```
